# Cytokine and Lymphocyte Profiles in COVID-19 Patients with Cancer: Implications for Disease Severity and Clinical Outcomes

**DOI:** 10.3390/v18070733

**Published:** 2026-07-02

**Authors:** Marina M. Burlá, Karina L. Silva, Bárbara C. Peixoto, Livia R. Goes, Isaclaudia Azevedo-Quintanilha, Fernando A. Bozza, Marcelo A. Soares, Andreia C. de Melo, Eugenio D. Hottz, Patricia T. Bozza, João P. B. Viola

**Affiliations:** 1 Program of Immunology and Tumor Biology, Brazilian National Cancer Institute (INCA), Rio de Janeiro 20231-050, RJ, Brazil; marinamburla@gmail.com (M.M.B.); karinalani@inca.gov.br (K.L.S.);; 2 Faculty of Medicine, Institute of Medical Education (IDOMED), Rio de Janeiro 20071-004, RJ, Brazil; 3 Program of Genetics and Tumor Virology, Brazilian National Cancer Institute (INCA), Rio de Janeiro 20231-050, RJ, Brazil; liviargoes@unirio.br (L.R.G.); masoares@inca.gov.br (M.A.S.); 4 Department of Genetics and Molecular Biology, Federal University of Rio de Janeiro State (UNIRIO), Rio de Janeiro 20211-010, RJ, Brazil; 5 Laboratory of Immunopharmacology, Oswaldo Cruz Institute (IOC), Oswaldo Cruz Foundation (FIOCRUZ), Rio de Janeiro 21040-361, RJ, Brazilpbozza@ioc.fiocruz.br (P.T.B.); 6 Intensive Care Medicine Laboratory, National Institute of Infectiology (INI), Oswaldo Cruz Foundation (FIOCRUZ), Rio de Janeiro 21040-361, RJ, Brazil; 7 D’Or Institute for Research and Education (IDOr), Rio de Janeiro 22281-100, RJ, Brazil; 8 Division of Clinical Research and Technological Development, Brazilian National Cancer Institute (INCA), Rio de Janeiro 20231-050, RJ, Brazil; 9 Laboratory of Immunothrombosis, Department of Biochemistry, Federal University of Juiz de Fora (UFJF), Juiz de Fora 36036-330, MG, Brazil

**Keywords:** cancer, COVID-19, T lymphocyte, NK cells, Treg cells, T-cell exhaustion, CXCL10/IP-10, MIF

## Abstract

Patients with cancer are at increased risk of severe outcomes from COVID-19. Yet, the immunological determinants underlying this vulnerability remain incompletely understood, particularly in low- and middle-income settings. Moreover, the impact of the severe viral disease surge and its compensatory mechanisms, such as stressed myelopoiesis, on this population needs further elucidation. This study aims to characterize the cytokine and lymphocyte profiles of cancer patients with COVID-19, correlate these profiles with disease severity, and compare them to those of non-cancer patients with COVID-19. Plasma cytokine, chemokine, and growth factor levels were quantified using Luminex technology, and immune cell subsets were characterized by flow cytometry. A total of 67 patients were analyzed: 40 with cancer (26 mild cases and 14 severe cases) and 27 without cancer (12 mild cases and 15 severe cases). Clinical outcomes showed an 86% mortality rate in cancer patients due to severe COVID-19. This contrasted with a 3.8% mortality rate in cancer patients with mild COVID-19, all unrelated to the infection. Our findings revealed elevated CXCL10 (IP-10) and reduced MIF levels in cancer patients with COVID-19, distinguished by disease severity. Compared with that in cancer patients with mild COVID-19, the level of CXCL10 in cancer patients with severe COVID-19 was further elevated. Additionally, cancer patients with COVID-19 presented reduced CD3^+^ T lymphocytes, expansion of CD4^+^CD25^+^FoxP3^+^ regulatory cells and CD56^BRIGHT^ NK cells, a shift from effector memory to central memory T-cells, and increased numbers of exhausted (PD-1^+^) T lymphocytes. In conclusion, our data suggest a distinct immunological profile observed in cancer patients with COVID-19. Especially in severe cases, viral surge-related suppressor cells and proinflammatory cytokines were accompanied by a compensatory immunosuppressive state, with decreased effector function and increased exhaustion. This may negatively impact clinical outcomes and highlight potential implications for the management of cancer patients.

## 1. Introduction

The COVID-19 pandemic exposed several challenges for public health systems worldwide, especially among patients with baseline diseases and comorbidities, such as cancer [1,2,3,4]. Compared to the general population, oncologic patients have higher mortality rates and more severe outcomes, either because of their underlying illness pathophysiology or because of their immunosuppressive drug regimen [2,4]. Thus, infection prevention and treatment optimization remain crucial in their management guidelines [3]. Furthermore, the rise of new strains and their high transmissibility are still concerns, even in the postvaccination era [3].

According to current evidence, severe COVID-19 cases may be associated with a depleted lymphocyte population, an exhausted T-cell profile, and elevated inflammatory cytokine levels [5,6,7]. Moreover, lymphopenia and decreased viral clearance were also associated with poorer prognosis in severe cases of acute respiratory infection. Investigations suggest that the rapid proliferation of immature myeloid-derived suppressor cells (MDSCs) plays a role in T-cell depletion, apoptosis, and overall systemic immunosuppression during severe disease [7,8]. By analyzing granulocytic and monocytic MDSCs, it is suggested that an initial surge of pro-inflammatory cytokines, such as IL-6 and G-CSF, triggers counterregulatory recruitment and expansion of these immunosuppressive clones from the bone marrow. Consequently, this could correlate directly with a significant decrease in CD4^+^ and CD8^+^ effector T-cells in severe COVID-19 patients, characterizing a compensatory state known as stressed myelopoiesis [8].

Elevated levels of PD-1, CD95, and apoptotic molecules have been associated with COVID-19 severity in cancer patients [7]. Even so, molecular and immunologic interactions between both conditions, and their implications for outcomes, remain unclear [7]. Therefore, this study aimed to evaluate the cytokine and cellular activation profiles of cancer patients with COVID-19. Moreover, we aimed to compare the cytokine and lymphocyte profiles of COVID-19 cancer patients with those of non-cancer patients and to correlate specific profiles with disease severity. Our data revealed that CXCL10 (IP-10) and MIF expression differed among COVID-19 patients with cancer, depending on disease severity. Cancer patients with severe COVID-19 presented reduced T lymphocytes, amplification of regulatory lymphocytes (T and NK cells), and exhausted immune profiles.

## 2. Materials and Methods

### 2.1. Study Design and Participants

This was a prospective cohort study of cancer patients with mild or severe COVID-19 and non-cancer patients with mild or severe COVID-19. Cancer patients were selected from electronic medical records, and data from inpatients admitted to the INCA (Instituto Nacional de Câncer, Rio de Janeiro, Brazil) from June 2020 to August 2020 were compiled. Hospital admissions occurred for COVID-19 symptoms or other medical reasons. Contact with positive COVID-19 patients and symptoms throughout hospitalization were also reported. Non-cancer patients with laboratory-confirmed SARS-CoV-2 and diagnosed COVID-19 within 72 h of ICU admission at three reference centers (Instituto Estadual do Cérebro Paulo Niemeyer, Hospital Copa Star and Leblon Campaign Hospital, all in Rio de Janeiro, Brazil) from April 2020 to August 2020 were included.

The diagnosis and stratification of COVID-19 patients were based on the WHO guidelines [9]. Diagnostic confirmation was defined by a positive result on a real-time reverse transcriptase polymerase chain reaction (RT-PCR) assay of nasal and oropharyngeal swab samples using the U.S. Centers for Disease Control and Prevention (CDC) reagents and protocol [10].

This study was approved by the Brazilian National Commission of Ethics in Research (approval number: CAAE 30608220.8.0000.5274 approved at 17 April 2020 and CAAE 30650420.4.1001.0008 approved at 19 April 2020) and was conducted following the Good Clinical Practice guidelines, keeping participant identities confidential.

### 2.2. Data Collection and Analysis

Demographic and clinical features, such as comorbidities, tumor subtype, tumor stage, metastatic sites, cause of death, and laboratory test results at diagnosis during hospitalization, were collected from the medical records. Clinical treatments for cancer and COVID-19 were also collected. Patients were classified as having severe COVID-19 in cases where mechanical ventilation and/or death occurred due to the infection. Patients with deaths unrelated to COVID-19 and not requiring mechanical ventilation due to the infection were not included in the severe group. Otherwise, patients were classified as having mild/moderate COVID-19. Patients who had not been discharged from the hospital were monitored for 28-day mortality.

We collected plasma from COVID-19 patients with or without cancer to evaluate their immunological profiles. We analyzed 42 different cytokines, chemokines, and growth factors in the plasma samples: IL-1α, IL-1β, IFN-α2, IL-6, IL-12, IL-12 (p40), IL-18, LIF, MIF, TNF-α, TNF-β, TRAIL, IL-2, IFN-γ, IL-4, IL-5, IL-13, IL-10, IL-15, IL-16, IL-17, IL-1Rα, IL-2Rα, IL-3, IL-7, IL-9, BASIC-FGF, βNGF, PDGF-BB, G-CSF, HGF, SCF, M-CSF, SCGFβ, MIP-1α, MIP-1β, MCP-1, MCP-3, IL-8, CTACK, RANTES, EOTAXIN, SDF1α, MIG, GROα, and IP-10. The cytokine levels were assessed by Luminex technology (Bio-Plex Workstation; Bio-Rad Laboratories, USA). The data were analyzed using software provided by the manufacturer (Bio-Rad Laboratories, Hercules, CA, USA).

Cell characterization was performed by flow cytometry and revealed the following cell populations: T lymphocytes, CD3^+^; CD4 T lymphocytes, CD3^+^CD4^+^; CD8 T lymphocytes, CD3^+^CD8^+^; T regulatory cells (T_REG_), CD3^+^CD4^+^CD25^+^CD127^−^; FoxP3^+^; NK cells, CD3-CD56^+^; CD4 T effector memory cells (CD4 T_EM_), CD3^+^CD4^+^CD62L^−^CD95^+^CCR7^−^; CD8 T effector memory cells (CD8 T_EM_), CD3^+^CD8^+^CD62L^−^CD95^+^CCR7^−^; CD4 T central memory cells (CD4 T_CM_), CD3^+^CD4^+^CD62L^+^CD95^+^CCR7^+^; CD8 T effector memory cells (CD8 T_CM_), CD3^+^CD8^+^CD62L^+^CD95^+^CCR7^+^; CD4 T exhausted cells (CD4 T_EX_), CD3^+^CD4^+^PD-1^+^; and CD8 T exhausted cells (CD8 T_EX_), CD3^+^CD8^+^PD-1^+^. The gating strategies are presented in Supplementary Figures S1–S5.

### 2.3. Peripheral Blood Samples

We collected a total of 67 peripheral blood samples from patients with (*n* = 40) or without cancer (*n* = 27). Among the cancer patients, 26 had mild/moderate COVID-19 symptoms, while 14 had severe disease. Among the noncancer patients, 12 were in the mild/moderate COVID-19 group, while 15 were in the severe group. Briefly, peripheral blood was collected into EDTA tubes, and the plasma was separated after 15 min of centrifugation at 400× *g* and stored at −80 °C for further cytokine analysis in Luminex. Blood was subsequently diluted in PBS (1:1), and mononuclear cells were isolated after Ficoll (Histopaque-1077, Sigma-Aldrich, Co., St. Louis, MO, USA) density gradient centrifugation (400× *g*, 30 min). PBMCs were then washed with PBS, followed by two washes with PBS + FBS (2%). The number of cells was counted, and cell viability was measured by the trypan blue exclusion method. Approximately 5 × 10^6^ cells/vial were frozen in FBS supplemented with 2% DMSO in a liquid nitrogen storage tank. At an appropriate time, frozen PBMCs were thawed in a 37 °C water bath, followed by two washes with 10 mL of RPMI 1640 supplemented with 10% FBS and 1% L-glutamine. The cells were counted again using trypan blue dye for viability analysis, washed with PBS, and resuspended in PBS supplemented with 2% BSA for flow cytometry analysis.

### 2.4. Flow Cytometry Analysis

Flow cytometry was used to characterize T-cell composition in COVID-19 patients with or without cancer, focused on T-cell activation/memory, exhaustion, and regulation. NK cell profile was also evaluated. For cell surface and intracellular staining, PBMCs were suspended in 20 µL of Fc block solution 2% in FACS buffer (PBS 1×, BSA1%) for 10 min. Then, for surface staining, cells were incubated for 30 min at room temperature using the following fluorochrome-conjugated anti-human antibodies from BD Biosciences: CD3-PerCP-Cy5.5 (clone UCHT1), CD4-APC-H7 (clone RPA-T4), CD8-PE-Cy7 (clone RPA-T8), CD197 (CCR7)-BB515 (clone 2-L1-A), CD95-PE-Cy7 (clone DX2), CD62L-PE (clone DREG-56), CD279 (PD-1)-BB515 (EH12.1), CD25-PE-Cy7 (clone M-A251), CD127-Alexa Fluor 647 (clone HIL-7R-M21) and CD56-PE-Cy7 (clone B159). For FoxP3 intracellular staining (FoxP3-Alexa Fluor 488, clone 259D/C7 from BD Biosciences), we used the Transcription Factor Buffer Set (BD Biosciences, San Diego, CA, USA) for fixation/permeabilization of cells, and the protocol was performed according to the manufacturer’s instructions. Viable cells were identified by exclusion using Fixable Viability Stain 780 (BD Biosciences). Samples were analyzed using a BD FACS Canto II Cytometer (BD Biosciences, San Jose, CA, USA) and FlowJo software version 10.6.1 (TreeStar, San Diego, CA, USA). Gating strategies were based on fluorescence-minus-one or negative controls (Supplementary Figures S1–S5).

### 2.5. Statistical Analysis

Statistical analysis was performed using GraphPad Prism software version 7. Nonparametric one-way ANOVA with Kruskal–Wallis multiple test correction was used for statistical analysis of the results. Differences with *p* < 0.05 were considered statistically significant.

## 3. Results

We analyzed data from a total of 67 patients diagnosed with COVID-19. Among these patients, 40 (59.7%) were cancer patients, and 27 (46.3%) were non-cancer patients. These groups were further divided into mild or severe cases of COVID-19 based on their need for mechanical ventilation and/or death due to infection. Baseline characteristics are detailed in Table 1. The median age in all four groups was greater than 45 years, and the most frequent comorbidities were hypertension (41.6–64.3%) and diabetes (16.6–42.1%) (Table 1). Adenocarcinoma was the most common tumor subtype in cancer patients (30.1%), and stage IV disease was prevalent in mild and severe cases (31.5% and 57.1%, respectively) (Table 1). Both groups showed metastatic spread, most commonly to the lungs. Among cancer patients with severe disease, 50% were receiving systemic palliative therapy, while 21.4% were managed with best supportive care.

In patients with cancer, COVID-19-related mortality reached 86% for severe cases and 3.8% for mild cases, which were unrelated to the infection (Table 1). Among patients without cancer, no deaths were reported for patients with mild COVID-19, but patients with severe COVID-19 had a 66% mortality rate due to infection (Table 1).

Individual-level characteristics of cancer patients with mild and severe COVID-19 are presented in Supplementary Tables S1 and S2. Regarding symptoms, over 85% of patients with severe COVID-19 presented with dyspnea or worsening ventilatory parameters requiring mechanical ventilation. Other frequent symptoms included fever, cough, and fatigue. In contrast, the mild COVID-19 cancer group presented varied and nonspecific complaints. Notably, while nausea, vomiting, and diarrhea were frequently reported, over 30% were asymptomatic. Dyspnea was substantially less prevalent than in the severe cohort (less than 20%). The severe COVID-19 group exhibited a higher proportion of patients with stage IV and stage II malignancies compared to those with mild COVID-19. Oncological management across the cohort was highly varied, encompassing chemotherapy, radiotherapy, surgical interventions, and palliative care. Most patients had initiated their cancer treatments before the onset of the study period, and most of the cohort was not under chronic corticosteroid therapy at the time of admission.

**Figure 1:**
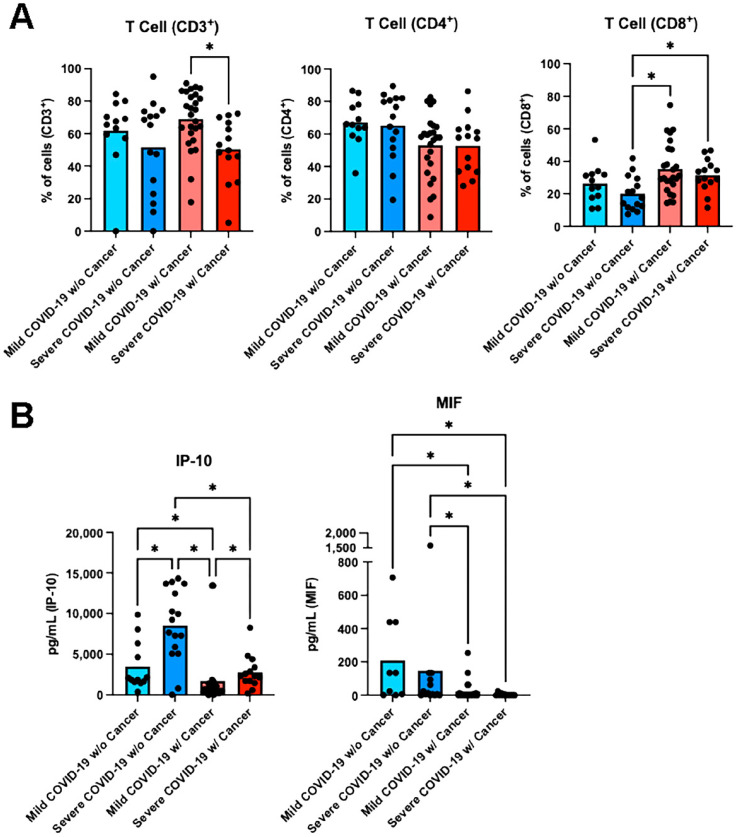
Analysis of T lymphocytes and cytokines from peripheral blood samples. Blood samples from hospitalized patients with mild or severe COVID-19 with and without cancer were analyzed. Each individual dot represents one patient, and the column represents the mean. (**A**) T lymphocyte analysis in each of the participants was performed by flow cytometry and revealed the presence of CD3^+^, CD3^+^CD4^+^ or CD3^+^CD8^+^ lymphocytes. (**B**) Analysis of cytokines (CXCL10/IP-10 and MIF) in each of the participants was performed by Luminex. Nonparametric one-way ANOVA with multiple comparisons and the two-stage step-up method of Benjamini, Krieger and Yekutieli were used for statistical analysis of the results. (*) *p* < 0.05 between selected groups.

In terms of T-cell characteristics, compared with cancer patients with severe COVID-19, cancer patients with mild COVID-19 presented increased T CD3^+^ populations (Figure 1A). In addition, considering only severe cases of COVID-19, the T CD8^+^ population was greater in cancer patients than in non-cancer patients (Figure 1A). The proportions of CD4^+^ T lymphocytes did not significantly differ between our participants (Figure 1A).

**Figure 2:**
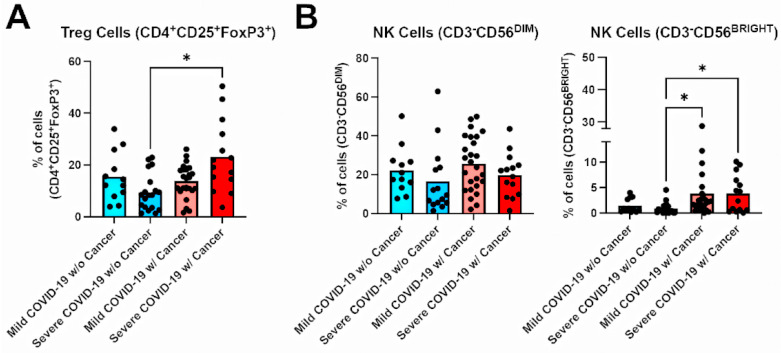
Analysis of Tregs and NK cells from peripheral blood samples. Blood samples from hospitalized patients with mild or severe COVID-19 with and without cancer were analyzed. Each individual dot represents one patient, and the column represents the mean. (**A**) T_REG_ from each of the participants were analyzed by flow cytometry for CD3^+^CD4^+^CD25^+^CD127^−^FoxP3^+^, and (**B**) NK cells were analyzed for CD3^−^CD56^+^. Nonparametric one-way ANOVA with multiple comparisons and the two-stage step-up method of Benjamini, Krieger and Yekutieli were used for statistical analysis of the results. (*) *p* < 0.05 between selected groups.

Analysis of cytokine levels in plasma revealed that the expression of chemokine CXCL-10 (IP-10) was increased in cancer patients compared with that in noncancer patients with COVID-19, both in mild cases and severe cases (Figure 1B). Compared with cancer patients with severe COVID-19, cancer patients with mild COVID-19 had lower CXCL-10 levels (Figure 2B). Furthermore, MIF expression was significantly lower in cancer patients with COVID-19 than in non-cancer patients (Figure 1B). However, our results revealed no significant differences between cancer patients and non-cancer patients in any of the other cytokine analyses.

**Figure 3:**
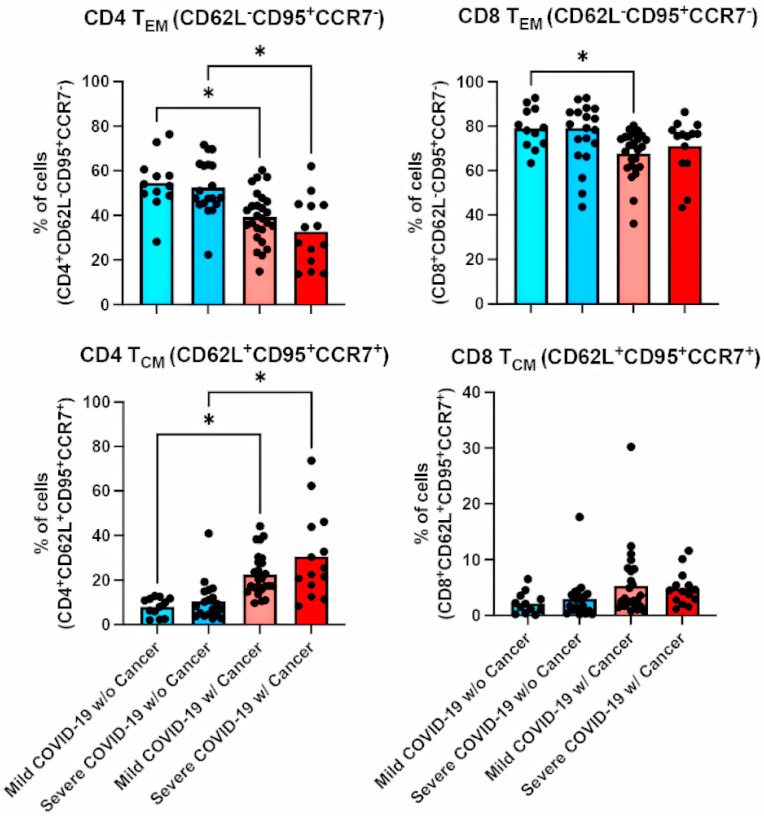
Analysis of memory cells from peripheral blood samples. Blood samples from hospitalized patients with mild or severe COVID-19 with and without cancer were analyzed. Each individual dot represents one patient, and the column represents the mean. Effector memory (EM) T lymphocyte analysis in each of the participants was performed by flow cytometry for CD3^+^CD4^+^CD62L^−^CD95^+^CCR7^−^ (CD4 T_CM_) or CD3^+^CD8^+^CD62L^−^CD95^+^CCR7^−^ (CD8 T_CM_). Analysis of central memory (CM) T lymphocytes for each of the participants was performed by flow cytometry for CD3^+^CD4^+^CD62L^+^CD95^+^CCR7^+^ (CD4 T_CM_) or CD3^+^CD8^+^CD62L^+^CD95^+^CCR7^+^ (CD8 T_CM_). Nonparametric one-way ANOVA with multiple comparisons and the two-stage step-up method of Benjamini, Krieger and Yekutieli were used for statistical analysis of the results. (*) *p* < 0.05 between selected groups.

We also analyzed different subsets of T regulatory (T_REG_) cells and NK cells (Figure 2). In patients with severe COVID-19, cancer patients presented higher proportions of T_REG_ cells (CD4^+^CD25^+^FoxP3^+^) compared with non-cancer patients (Figure 2A). No differences were observed in the NK cell lineage CD3^+^CD56^DIM^ (Figure 2B). However, the CD3-CD56^BRIGHT^ population was significantly greater in cancer patients with severe COVID-19 than in non-cancer patients (Figure 2B). Furthermore, memory T-cell analysis (central and effector memory) revealed that compared with non-cancer patients, cancer patients with COVID-19 presented lower proportions of effector memory cells and higher numbers of central memory cells (Figure 3).

**Figure 4:**
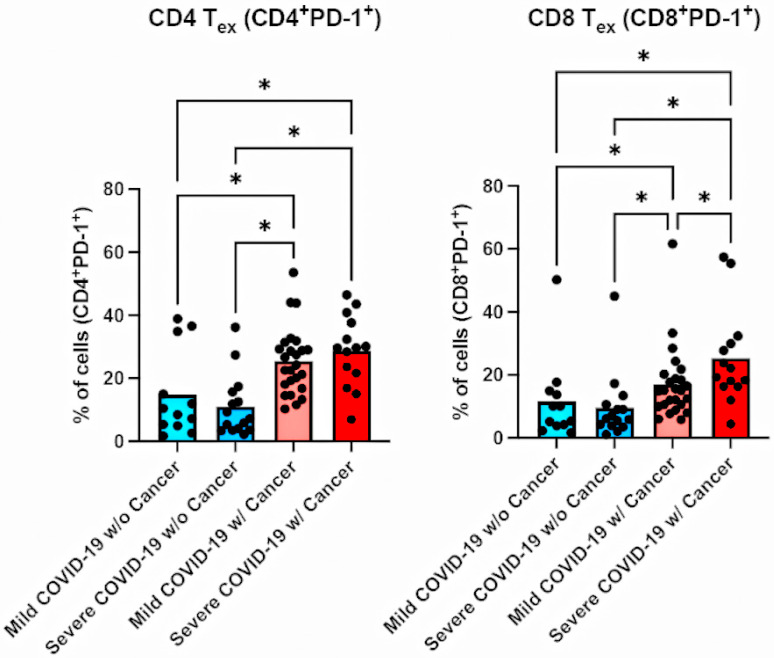
Analysis of exhausted T lymphocytes from peripheral blood samples. Blood samples from hospitalized patients with mild or severe COVID-19 with and without cancer were analyzed. Each individual dot represents one patient, and the column represents the mean. Exhausted (EX) T lymphocytes from each of the participants were analyzed by flow cytometry for CD3^+^CD4^+^PD-1^+^ (CD4 T_EX_) or CD3^+^CD8^+^PD-1^+^ (CD8 T_EX_). Nonparametric one-way ANOVA with multiple comparisons and the two-stage step-up method of Benjamini, Krieger and Yekutieli were used for statistical analysis of the results. (*) *p* < 0.05 between selected groups.

Finally, we analyzed the T-cell exhaustion profiles of cancer and non-cancer patients (Figure 4). Among mild COVID-19 patients, cancer patients had increased numbers of exhausted CD4 (CD4^+^PD-1^+^) and CD8 (CD8^+^PD-1^+^) T-cells compared with non-cancer patients (Figure 4). Similarly, among severe COVID-19 cases, cancer patients also had increased proportions of exhausted T-cells (CD4^+^PD-1^+^ and CD8^+^PD-1^+^) compared with non-cancer patients (Figure 4).

## 4. Discussion

Individuals who have comorbidities and are infected with COVID-19 face higher mortality rates and poorer outcomes, such as longer hospital stays and mechanical ventilation requirements [11]. This impact is particularly significant in cancer patients, who had an estimated mortality rate of 6% in 2020 [12]. Although these worsened outcomes have been attributed to immunosuppressive treatments and comorbidities, the underlying cytokine and lymphocyte activation profiles are being investigated to explain the poorer outcomes in oncologic patients [13,14,15,16,17].

The contrast in clinical symptoms observed in our cohort supports the presence of a distinct, virally driven systemic immune response [8,18,19]. The severe cancer cohort predominantly presented with respiratory symptoms, such as acute dyspnea and the need for mechanical ventilation. In severe acute respiratory viral infections, this aggressive lung injury and the subsequent release of pro-inflammatory cytokines could trigger ‘stressed myelopoiesis’, with MDSCs acting to mitigate massive tissue damage [8,19]. Similarly, severe clinical symptoms of respiratory distress observed in our patients could be linked to a compensatory immunosuppressive state: depletion of effector memory T-cells, amplification of regulatory cells, and heightened T-cell exhaustion. These clinical and immunological findings demonstrate how the severe viral surge overrides normal immune surveillance, driving a stressed myelopoiesis state that can ultimately worsen patient outcomes.

Our analysis revealed a significant association between CD3^+^ T-cell depletion and the severity of COVID-19, which could minimize an appropriate antiviral response. Although patients with severe COVID-19 presented with increased percentages of CD8^+^ T-cells, this population likely exhibits impaired function, marked by increased exhaustion markers and prominent regulatory T-cells within this subset [8,20,21,22,23,24,25]. Specifically, our results revealed a greater population of regulatory T-cells in cancer patients with severe COVID-19 than in non-cancer patients. Furthermore, our findings indicate a positive correlation between cancer and the severity of COVID-19 with increased levels of the exhaustion marker PD-1. These findings suggest that T-cell activation is suppressed during COVID-19 infection and that regulatory and exhaustion activity is increased, potentially impairing the antiviral activity of lymphocytes [26,27].

Additionally, NK cells are involved in the innate system response against viral infections and influence the tumor microenvironment [23]. Our results revealed higher rates of immature CD56^BRIGHT^ NK cells in cancer patients than in non-cancer patients with COVID-19 (Figure 2). This further reinforces a stressed myelopoiesis state, and progression of proinflammatory cytokine release instead of mature cytotoxic NK function [24]. However, the precise role of these cells in COVID-19 needs further investigation, especially to elucidate their antiviral activity in oncologic patients [23,24,25].

The systemic inflammatory response is also marked by specific cytokine alterations. The chemokine CXCL-10 (IP-10) correlates with the initial cytokine storm cascade in inflammatory states [8]. Our study found a positive association between CXCL-10 levels and disease severity in COVID-19 patients, which may reflect a shift toward an adaptive immune response. In contrast, although high MIF levels typically drive an immunosuppressive tumor microenvironment, our findings of lower MIF levels in cancer patients may point to a distinct exhaustion of the innate inflammatory response in this population [8,18,19,20,21].

This study has limitations. The relatively small number of patients may limit the generalizability of the findings. Because of this and the considerable heterogeneity in our cohort, we could not perform subgroup analyses based on tumor types or treatment strategies. Since the study was conducted at a single center, the results may not be representative of broader populations. On the other hand, the single-centered nature of our study warranted comparisons of patients who received clinical evaluation and COVID-19 and cancer standardized care following homogeneous international guidelines. Additionally, all patients were treated during the early stages of the COVID-19 pandemic, prior to the availability of vaccinations, which could influence disease outcomes and management strategies. These factors should be considered when interpreting the results, and further multicenter studies with larger cohorts in the postvaccination era are warranted to validate our findings.

## 5. Conclusions

Our results highlight the importance of assessing the lymphocyte activation profile in cancer patients with COVID-19. Understanding the alterations in specific T-cell subsets can provide insights into the immune response and potential immune dysregulation in this population. While our study has several limitations, our findings suggest that COVID-19 patients with cancer present amplification of regulatory lymphocytes, such as T and NK cells, along with an exhausted immune profile, which could impact cancer and COVID-19 clinical outcomes. Furthermore, COVID-19 patients with cancer exhibited a shift from effector memory to central memory T-cells. Additionally, we observed a positive association between CXCL-10 (IP-10) levels and disease severity in COVID-19 patients. Rather than conclusively establishing predictive biomarkers or mechanistic relationships, our data provide early immunological insights that serve as a foundation for future, larger-scale studies to validate these potential targets for therapeutic strategies.

## Figures and Tables

**Table 1:** Baseline Characteristics of Cancer Patients with COVID-19.

	Cancer Patients		Non-Cancer Patients	
	Mild COVID-19	Severe COVID-19	Mild COVID-19	Severe COVID-19
N of patients	26	14	12	15
Gender M/F (%)	30/70	36/64	50/50	53/47
Median age (range)	58.5 (18–78)	62 (29–78)	58 (33–80)	47 (26–93)
Comorbidities	Diabetes: 26.9% HT: 57.7% Obesity: 11% CF/CMP: 7.7% Others: 15.3%	Diabetes: 21.4% HT: 64.3% Obesity: 7.1% CF/CMP: 21.4% Others: 35.7%	Diabetes: 16.6% HT: 41.6% Obesity: 41.6% Others: 16.6%	Diabetes: 46.6% HT: 46.6% Obesity: 6.6% CF/CMP: 13.3%
Tumor subtype	AdenoCA: 26% SCC: 7.6% Sarcoma: 3.8% Others: 54% Missing: 8.6%	AdenoCA: 36% SCC: 21% Others: 36% Missing: 7%	N/A	N/A
Stage	I: 7.7% II: 7.7% III: 19.2% IV: 38.5% N/A: 30.8% ^#^ Missing: 3.8%	I: 7.1% II: 14.2% IV: 57.1% N/A: 14.3% ^##^ Missing: 7.1%	N/A	N/A
Metastatic sites *	Lungs: 11.5% Liver: 3.85% CNS: 3.5% Bones: 15.4% Lymph nodes: 3.9% Skin: 7.7%	Lungs: 21.4% Liver: 14.3% CNS: 14.3% Bones: 7.14% Lymph nodes: 14.3% Peritoneum: 7.14%	N/A	N/A
Intention to treat **	Adjuvant: 7.7% Neoadjuvant: 15.4% Palliative: 19.2% Curative: 19.2% Control: 15.4% Support: 19.2%	Adjuvant: 4.14% Palliative: 50% Curative: 7.14% Control: 21.4% Support: 21.4%	N/A	N/A
Deaths; Cause	3.8%; unrelated to COVID-19	86%; COVID-19	N/A	66%; COVID-19

N: number; F: female; M: male; HT: hypertension; CF: cardiac failure; CMP: cardiomyopathies; AdenoCA: adenocarcinoma; SCC: squamous cell carcinoma; CNS: central nervous system; N/A: not applicable. ^#^ TNM staging was not applicable to 5 cases, including multiple myeloma (*n* = 2), myelodysplastic syndrome/acute myeloid leukemia (*n* = 1), chronic myeloid leukemia in lymphoid blast crisis (*n* = 1), and acute lymphoblastic leukemia (*n* = 1). ^##^ TNM staging was not applicable to 1 case of multiple myeloma. One additional case of leukocytosis under evaluation did not have a definitive diagnosis and was therefore not staged. * Multiple metastatic sites were observed in some patients. More than one category may apply. Palliative treatment was stratified into first-, second-, and third-line therapy. ** Supportive treatment refers to best supportive care. Control treatment refers to maintenance therapy.

## Data Availability

The data generated in the present study may be requested from the corresponding author.

## Supplementary Materials

The following supporting information can be downloaded at: https://www.mdpi.com/article/10.3390/v18070733/s1, Figure S1. Representative flow cytometry gating strategy for identification of T lymphocyte subsets (CD4+ and CD8+) in PBMCs from a representative sample. Figure S2. Flow cytometric analysis and gating hierarchy for regulatory T cells (Treg) from a representative sample. Figure S3. Gating strategy for the characterization of Natural Killer (NK) cell subpopulations from a representative sample. Figure S4. Flow cytometry gating strategy for the identification of memory T cell subsets from a representative sample. Figure S5. Gating strategy for the evaluation of Programmed Cell Death Protein 1 (PD-1) expression on T cell subsets from a representative sample. Table S1. Individual-level data of cancer patients with mild COVID-19. Table S2. Individual-level data of cancer patients with severe COVID-19.

## Abbreviations

The following abbreviations are used in this manuscript:

ANOVA	Analysis of Variance
BSA	Bovine Serum Albumin
CDC	Centers for Disease Control and Prevention
COVID-19	Coronavirus Disease 2019
CXCL10	C-X-C Motif Chemokine Ligand 10
DMSO	Dimethyl Sulfoxide
FBS	Fetal Bovine Serum
IFN	Interferon
ICU	Intensive Care Unit
IL	Interleukin
INCA	Instituto Nacional do Câncer
IP-10	Interferon Gamma-Induced Protein 10
MIF	Macrophage Migration Inhibitory Factor
NK	Natural Killer
PBS	Phosphate-Buffered Saline
PD-1	Programmed Cell Death Protein 1
PBMC	Peripheral Blood Mononuclear Cells
RT-PCR	Reverse Transcription Polymerase Chain Reaction
SARS-CoV-2	Severe Acute Respiratory Syndrome Coronavirus 2
TCM	Central Memory T-cells
TEX	Exhausted T-cells
TIM-3	T-cell immunoglobulin and mucin-domain containing-3
TNF	Tumor Necrosis Factor
TREG	Regulatory T-cells
TEM	Effector Memory T-cells
WHO	World Health Organization
