# Supplementary material for: Cytokine and Lymphocyte Profiles in COVID-19 Patients with Cancer: Implications for Disease Severity and Clinical Outcomes
Source: Viruses. 2026 Jul 2;18(7):733. doi: 10.3390/v18070733 (PMC13431523; doi:10.3390/v18070733)
Supplement: Supplementary file 1 [file viruses-18-00733-s001.zip › viruses-4320453-supplementary.pdf]

Supplementary Table S1. Individual-level data of cancer patients

	Chronic use of corticosteroids	Diagnosis	Current Staging	Current treatment	Date first treatment	Date last treatment (<60 days prior)	Symptoms
**Patients with Mild COVID**							
ID 15	No	Plasmablastic lymphoma	IV	Chemotherapy	March, 2020	June, 2020	No symptoms
ID 16	No	Pancreatic cancer (Adenocarcinoma)	III	Palliative care	June, 2020	NA	No symptoms
ID 17	No	Non-Hodgkin lymphoma	II	Chemotherapy	May, 2020	June, 2020	Fever, diarrhea, dyspnea, nausea, vomiting, anorexia
ID 18	No	Chronic myeloid leukemia (CML) with blast crisis	NA	Chemotherapy, Targeted therapy	January, 2020	April, 2020	Dyspnea, fatigue
ID 19	NA	Cutaneous squamous cell carcinoma (SCC)	IV	Palliative care	NA	NA	Cough, dyspnea, fatigue, diarrhea
ID 20	No	Cervical cancer (Adenocarcinoma)	I	Follow-up	2013	NA	No symptoms
ID 21	No	Biliary tract cancer (Adenocarcinoma)	IV	Surgery, Chemotherapy	June, 2020 / June, 2020	June, 2020	Fever
ID 22	Yes	Multiple myeloma	NA	No treatment to date	NA	NA	Fever, cough, dyspnea, fatigue, myalgia, diarrhea, nausea, vomiting
ID 23	No	Breast cancer	III	Chemotherapy	March, 2020	June, 2020	Fever, diarrhea
ID 24	Yes	Myelodysplastic syndrome/Acute myeloid leukemia (MDS/AML)	NA	Chemotherapy	July, 2020	July, 2020	Headache, nausea, vomiting
ID 25	No	Osteosarcoma	IV	Surgery	June, 2020	June, 2020	No symptoms
ID 26	No	Leiomyosarcoma	IV	Radiotherapy	December, 2019	NA	Fever, cough, dyspnea, fatigue
ID 27	No	Shoulder mass under investigation	NA	No treatment to date	NA	NA	Cough, dyspnea
ID 28	No	Prostate cancer (Adenocarcinoma)	I	Hormonal therapy	November, 2018	July, 2020	Nausea, vomiting, anorexia
ID 29	No	Colon cancer (Adenocarcinoma)	II	Surgery	June, 2020	June, 2020	Fever, cough, dyspnea
ID 30	No	Prostate cancer (Adenocarcinoma)	IV	Hormonal therapy, Chemotherapy	February, 2020	May, 2020 (Zoladex)	No symptoms
ID 31	No	Cervical cancer (SCC)	III	Follow-up	June, 2019	NA	Fatigue, nausea, vomiting
ID 32	No	Endometrial cancer (Adenocarcinoma)	IV	Follow-up	February, 2020	NA	No symptoms
ID 33	No	Breast cancer	IV	Hormonal therapy	July, 2020	Continuous	No symptoms
ID 34	No	Chronic myeloid leukemia (CML) with lymphoid blast crisis	NA	Chemotherapy, Targeted therapy	March, 2020	July, 2020	Fever, cough, dyspnea, fatigue, anosmia, diarrhea, nausea, vomiting, anorexia
ID 35	No	Multiple myeloma	NA	Chemotherapy, Targeted therapy	January, 2019	July, 2020	Fever, cough, headache
ID 36	No	Breast cancer	III	Chemotherapy	July, 2020	July, 2020	No symptoms
ID 37	No	Acute lymphoblastic leukemia (ALL)	NA	No treatment to date	NA	NA	Fatigue, cough, nausea, vomiting, anorexia
ID 38	No	Diffuse Large B-Cell Lymphoma	IV	Chemotherapy	April, 2020	July, 2020	No symptoms
ID 39	No	Cervical cancer (SCC)	III	Surgery	August, 2020	August, 2020	NA
ID 40	No	Breast cancer	IV	Chemotherapy	March, 2020	August, 2020	No symptoms

ALL: acute lymphoblastic leukemia; AML: acute myeloid leukemia; CA: carcinoma (cancer); CML: chronic myeloid leukemia: MDS: myelodysplastic syndrome; MDS/AML: myelodysplastic syndrome/acute myeloid leukemia; NA: not available/not applicable; SCC: squamous cell carcinoma.

Supplementary Table S2. Individual-level data of cancer patients

	Chronic use of corticosteroids	Diagnosis	Current Staging	Current treatment	Date first treatment	Date last treatment (<60 days prior)	Symptoms
**Patients with Severe COVID**							
ID 1	No	Hepatocellular carcinoma	I	Surveillance only	December, 2019	NA	Dyspnea, cough, myalgia, anorexia
ID 2	No	Breast cancer	IV	Hormonal therapy; Surgery	October, 2018	Continuous use; Surgery June, 2020	No symptoms
ID 3	Yes	Colon cancer (Adenocarcinoma)	IV	Chemotherapy	December, 2019	March, 2020	Dyspnea
ID 4	No	Endometrial cancer (Adenocarcinoma)	IV	Surgery	June, 2020	June, 2020	Worsening ventilatory parameters during mechanical ventilation
ID 5	No	Prostate cancer (Adenocarcinoma)	II	Surgery	June, 2020	June, 2020	Dyspnea, fever
ID 6	No	Endometrial cancer (Adenocarcinoma)	NA	Palliative care	June, 2020	NA	Dyspnea
ID 7	NA	Breast cancer	IV	Hormonal therapy	November, 2018	Continuous	Dyspnea
ID 8	No	Leukocytosis without a hematologic diagnosis	NA	Chemotherapy	July, 2020	July, 2020	Cough, dyspnea, fatigue
ID 9	No	Colon cancer (Adenocarcinoma)	IV	Chemotherapy	February, 2020	July, 2020	Fever, cough, dyspnea
ID 10	No	Lung cancer (SCC)	IV	Chemotherapy	May, 2020	July, 2020	Cough, dyspnea, fatigue
ID 11	No	Hodgkin lymphoma	II	6	1995	NA	Dyspnea
ID 12	No	Lung cancer (SCC)	IV	Chemotx, Radiotherapy	May, 2020	July, 2020	Fever, cough, dyspnea
ID 13	NA	Multiple myeloma	NA	Chemotherapy, Targeted therapy	July, 2019	July, 2020	Fever, cough, fatigue, diarrhea, nausea, vomiting
ID 14	No	Cervical cancer (SCC)	IV	Palliative care	February, 2020	NA	Fever, dyspnea, fatigue, nausea, vomiting

ALL: acute lymphoblastic leukemia; AML: acute myeloid leukemia; CA: carcinoma (cancer); CML: chronic myeloid leukemia: MDS: myelodysplastic syndrome; MDS/AML: myelodysplastic syndrome/acute myeloid leukemia; NA: not available/not applicable; SCC: squamous cell carcinoma.

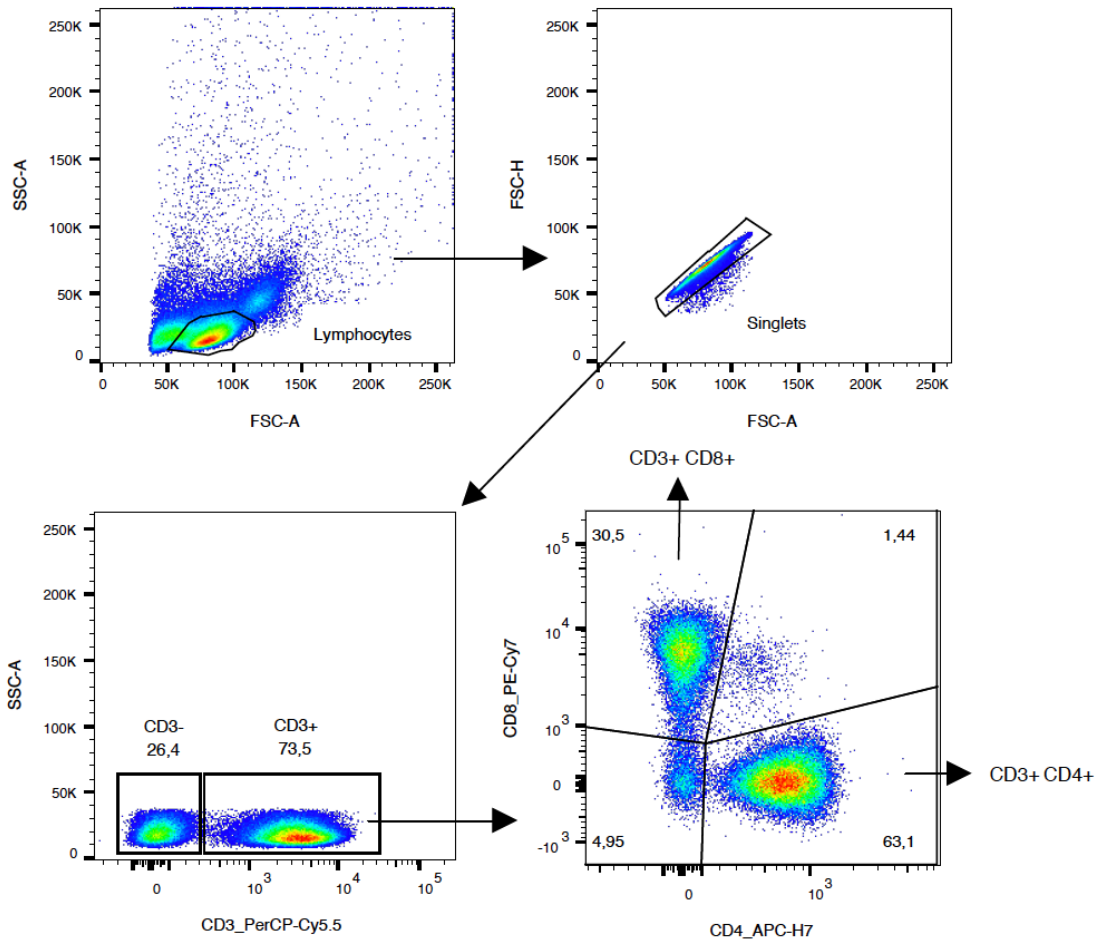

Supplementary Figure S1. Representative flow cytometry gating strategy for identification of T lymphocyte subsets ($CD4^{+}$ and $CD8^{+}$) in PBMCs from a representative sample. Lymphocytes were initially identified and gated based on their forward scatter (FSC-A) and side scatter (SSC-A) properties (Top left). Doublets were excluded by gating on single cells (Singlets) using FSC-A versus FSC-H parameters (Top right). Total T cells were identified by the expression of CD3 (CD3_PerCP-Cy5.5) within the singlet population (Bottom left). Subsequently, within the $CD3^{+}$ gated T cells, helper T cells $CD4^{+}$ (CD4_ APC-H7) and cytotoxic T cells $CD8^{+}$ (CD8_PE-Cy7) were quantified and discriminated (Bottom right). Numbers inside graphs indicate the percentage of cells within each respective gate.

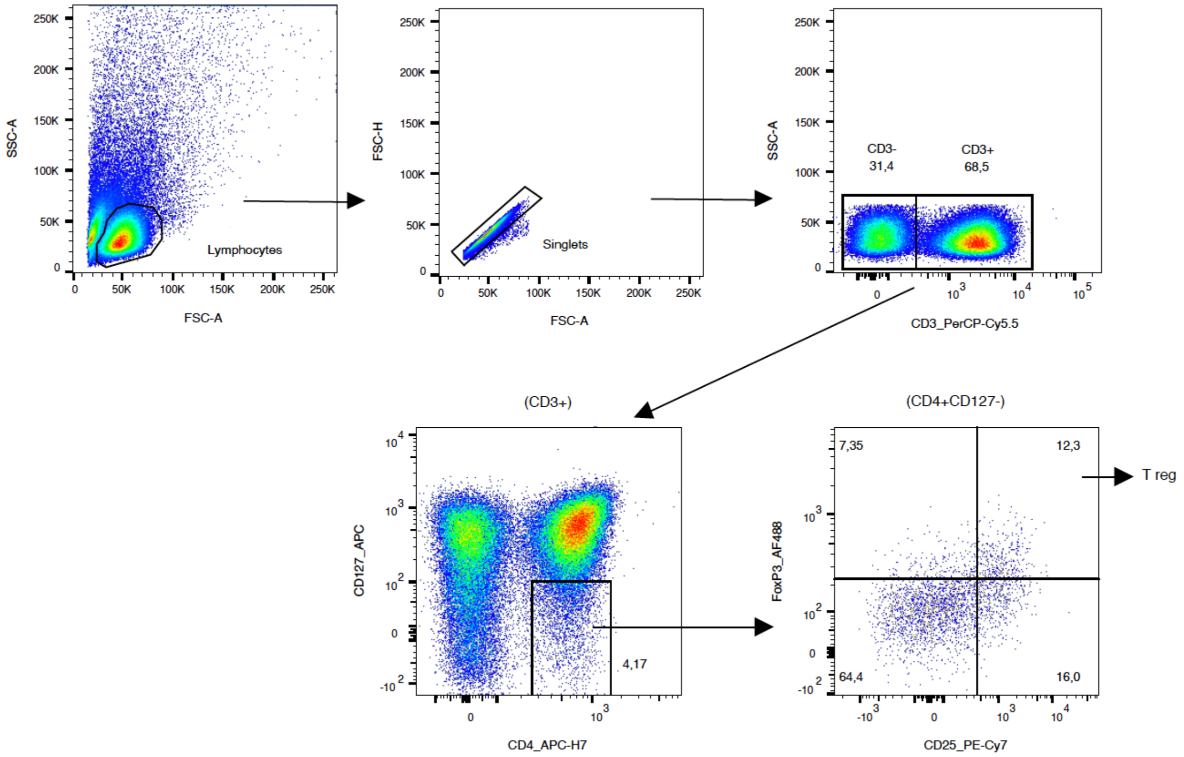

Supplementary Figure S2. Flow cytometric analysis and gating hierarchy for regulatory T cells (Treg) from a representative sample. Sequential gating was applied starting from the lymphocyte population (FSC-A vs. SSC-A) (Top left) and doublet discrimination (FSC-A vs. FSC-H) (Top middle). Total T cells were selected based on CD3 expression ($CD3^+$ PerCP-Cy5.5) (Top right). From the $CD3^+$ population, the $CD4^+CD127^-$ subset was identified (CD127_APC vs. CD4_APC-H7) (Bottom left). Finally, regulatory T cells (Treg) were defined within the $CD4^+CD127^-$ fraction by the co-expression of CD25_PE-Cy7 and the intracellular transcription factor FoxP3_AF488 (Bottom right). Numbers represent the percentages of gated cells.

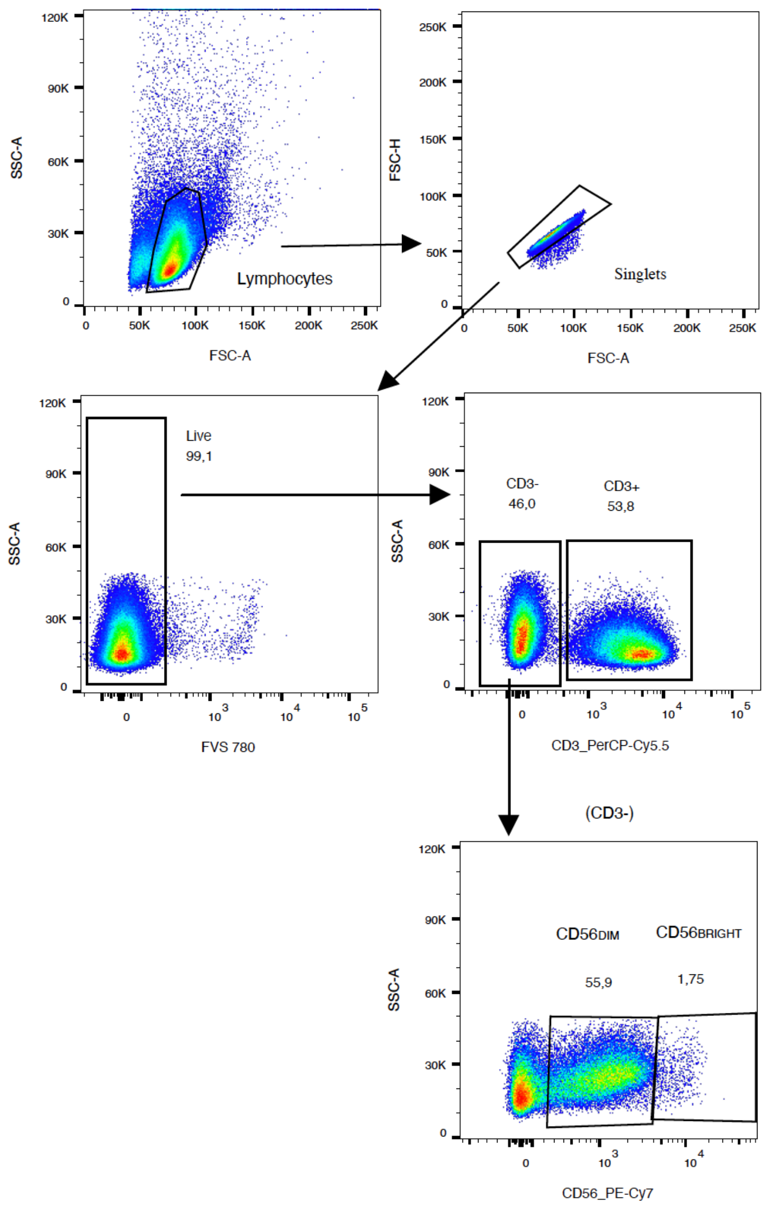

Supplementary Figure S3. Gating strategy for the characterization of Natural Killer (NK) cell subpopulations from a representative sample. Initial selection of lymphocytes (FSC-A vs. SSC-A) and single cells (FSC-A vs. FSC-H) was performed. Viable cells were identified by negative staining for the viability dye Fixable Viability Stain 780 (FVS 780). Within the live cell population, $CD3^-$ cells were gated (CD3_PerCP-Cy5.5 vs. SSC-A). From the $CD3^-$ population, NK cell subsets were characterized according to CD56 expression density (CD56_PE-Cy7), distinguishing between $CD56^{DIM}$ and $CD56^{BRIGHT}$ NK cells. Percentages of cells in each gate are indicated.

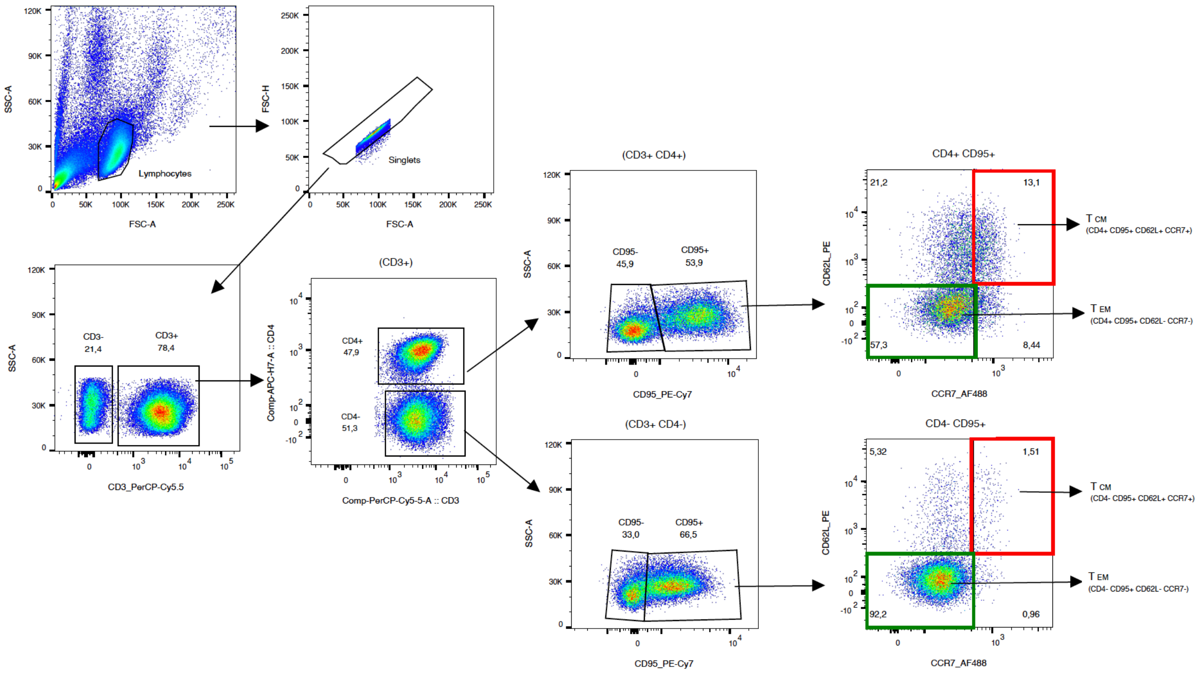

Supplementary Figure S4. Flow cytometry gating strategy for the identification of memory T cell subsets from a representative sample. Following lymphocyte selection, doublet exclusion, and total T cell tracking (CD3_PerCP-Cy5.5), $CD3^{+}$ cells were segregated into $CD4^{+}$ (CD4_APC-H7) and $CD4^{-}$ ($CD8^{+}$) T cell populations. Both $CD4^{+}$ and $CD4^{-}$ fractions were analyzed for the expression of the activation/memory marker CD95 (PE-Cy7). Within the $CD95^{+}$ memory compartments, Central Memory T cells (T_{CM}) and Effector Memory T cells (T_{EM}) were further dissected based on the expression profiles of CD62L (PE) and CCR7 (AF488): T_{CM} were defined as $CD62L^{+}CCR7^{+}$ and T_{EM} as $CD62L^{-}CCR7^{-}$. Numbers indicate the percentage of cells within each quadrant/gate.

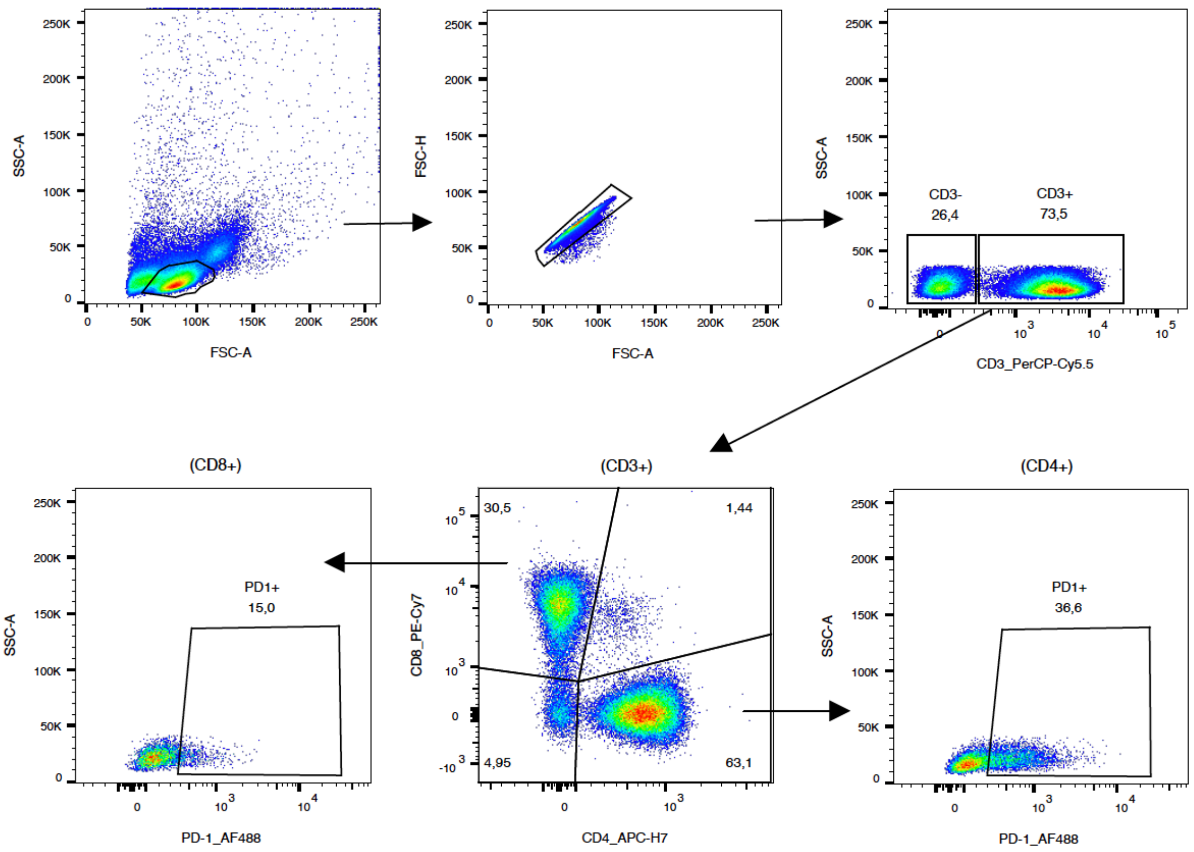

Supplementary Figure S5. Gating strategy for the evaluation of Programmed Cell Death Protein 1 (PD-1) expression on T cell subsets from a representative sample. Lymphocytes were sequentially gated for single cells and total $CD3^{+}$ T cells (CD3_PerCP-Cy5.5). Total T cells were separated into cytotoxic $CD8^{+}$ T cells (CD8_PE-Cy7) and helper $CD4^{+}$ T cells (CD4_APC-H7). PD-1 expression (AF488) was independently evaluated on both $CD8^{+}$ (left) and $CD4^{+}$ (right) T cell gates to determine the frequency of PD-1^{+} cells. Numbers represent the percentage of positive cells within the respective gates.
